# Commentary: Addictions Neuroclinical Assessment: A Neuroscience-Based Framework for Addictive Disorders

**DOI:** 10.3389/fpsyt.2017.00002

**Published:** 2017-01-13

**Authors:** Udi E. Ghitza

**Affiliations:** ^1^National Institute on Drug Abuse Center for Clinical Trials Network, Bethesda, MD, USA

**Keywords:** addiction, addictive disorders, addiction treatment, drug abuse, drug abuse treatment, personalized medicine, precision medicine, substance abuse

Kwako et al. recently proposed a neuroscience-based framework by which to classify substance use disorders (SUDs) (1). This is based on functional domains—incentive salience, negative emotionality, and executive function—derived from a cycle-of-addiction model. The authors provide a thoughtful literature synthesis as rationale for an associated Addictions Neuroclinical Assessment (ANA) aiming to accelerate precision medicine research on relationships between genetic by environmental-exposure interactions and phenotypic expression of these SUD domains. Precision medicine may be defined as “an emerging approach for disease treatment and prevention that takes into account individual variability in genes, environment, and lifestyle for each person” (2). The authors explain how ANA may guide multidimensional analyses informing how to customize SUD research and care to address cross-population and temporal variability in genetic and phenotypic expression of these domains tailored to different patient subgroups (1).

The introduction of a standardized neuroscience-based SUD assessment battery to advance precision medicine research is innovative and critical, and nicely complements similar efforts advanced by the U.S. National Institute of Mental Health’s Research Domain Criteria initiative (3–6). Utilizing standardized instruments for measuring core functional domains of SUD enables comparability across research studies, meta-analyses, and data mining to advance biomedical big-data research. However, improved standardized measurement on its own may not lead to translation of neuroscience-based research into better SUD care. For this to happen, research needs to incorporate two factors. First, studies are needed to validate how such domains may be used to tailor treatments to different levels of impairment and to test specificity and sensitivity of proposed ANA domains to SUD-related impairment. Study designs should also provide controls to account for the possible confounds in which participants who choose to frequently use alcohol or drugs might also have other co-occurring problems, either naturally or due to other lifestyle choices or circumstances. Target and biomarker validation—critical to expedite precision medicine research—necessitates such analyses.

Second, a concise assessment battery is essential to improve ease of use in deep-phenotyping efforts. Kwako et al.’s assessment battery is proposed to take a full 10 h to administer (1), which may place heavy burden on clinical researchers and most SUD patients—thus introducing a sample selection bias and compromising validity. A neuroscience-based nosology should be proposed with more precise functional domains, measured using a streamlined assessment battery of validated instruments. To shorten the battery and enhance its feasible administration across clinical and research situations, modifiable assessments are necessary, tailored to risk categories. These could be based upon a shared decision-making approach where research participants are queried on functional domains most impeding their overall well-being. Such modifiable assessments would shorten the ANA, by incorporating only those phenotypic measurements deemed to be most relevant to functioning of particular participants.

More rigorous research is needed to systematically evaluate a patient-centered, neuroscience-based framework for treating SUDs—testing a shared decision-making, precision medicine approach tailored to salient risk categories of each participant. Pre-dating the ANA, in 2014, I developed an approach, termed the ASPIRE model (7, 8), that uses as its foundational principle shared decision making between patients and providers to tailor personalized medical care and precision medicine research to six neuroscience-based risk categories in which individual patients report as most distressing to their daily lives. The ASPIRE-framework risk categories—proposed based on over 30 years of neuroscience research and representing common pathophysiological factors related to etiology and perpetuation of substance use-related problems—include the following. (A) *A*nhedonia/reward-deficit state; (S) *S*tressful/anti-reward state; (P) *P*athological lack of self-control to cut down substance use despite undesired consequences; (I) *I*nsomnia associated with substance use and worsening functional impairment; (R) *R*estlessness associated with the above; (E) *E*xcessive preoccupation with seeking drug reinforcers, compared with natural reinforcers, following transition from volitional to compulsive drug use, particularly associated with craving on exposure to a drug-associated environment (7, 8). The precise meaning of these empirically derived constructs is subjected to change in the basis of ongoing research.

Table 1 presents a proposal for a more succinct assessment battery of validated standardized instruments that map onto these risk categories. This could serve as a model for phenotyping purposes and to advance biomedical big-data science using standardized data collection, which permits pooling data for SUD precision medicine research. Assessment battery of Table 1 contains relevant PROMIS^®^ (Patient-Reported Outcomes Measurement Information System) measures (9). These have been developed and validated with U.S. National Institutes of Health funding, utilizing state-of-the-science item response theory and other statistical methods to be psychometrically sound. These measures are intended to comprise a brief assessment battery, which would be administered using computer adaptive tests containing skip patterns where they would *only* be presented to participants if they assess risk categories participants report as being most pertinent to their functional impairments. Otherwise, these questions would be omitted, which would substantially shorten length of administration and minimize burden on researchers and participants alike, thereby enhancing feasibility and ecological validity to most research and clinical situations. This proposed question set also contains Core Tier-1 measures of Substance Abuse and Addiction project of the phenotypes and exposures (PhenX) Toolkit, and Core Tier-2 measures whenever possible and appropriate. The U.S. National Institute on Drug Abuse (NIDA) strongly encourages all NIDA supported human subjects researchers to use standardized measures to facilitate measure commonality and data comparability (10). Notably, all suggested measures are non-proprietary and open-source, which could broaden use by minimizing cost. Use of data standards a means to promote cross-study comparisons and combined data analyses needed to validate and extend human subjects research results.

**Table 1:** **Proposed measures: computer-based assessment with skip patterns to shorten length**.

Adult PROMIS^®^ domain^a^ http://www.healthmeasures.net/explore-measurement-systems/promis/intro-to-promis/list-of-adult-measures	Short forms *up to* a maximum^d^ items (below), depending on research question	Computer adaptive tests (CAT) item banks^e^ maximum^d^ items *(asked based on responses)*	ASPIRE model risk category mapping to adult PROMIS^®^ domain. For more information on ASPIRE problem categories, see Ghitza (7, 8)^f^
Emotional distress—depression	8	30	(A) Anhedonia/reward-deficit state
Emotional distress—anxiety	8	29	(S) Stressful/anti-reward state
Pain interference	8	40	(S)
Alcohol—negative expectancies^b^	7	11	(S)
Alcohol—negative consequences^b^	7	31	(S)
Smoking—negative health expectancies^c^	6	19	(S)
Smoking—coping expectancies^c^	4	18	(S)
Smoking—negative psychosocial expectancies^c^	6	20	(S)
Cognitive function	8	32	(P) Pathological lack of self-control to cut down substance use
Sleep-related impairment	8	16	(I) Insomnia associated with substance use/impairment; (R) Restlessness
Alcohol—positive expectancies^b^	7	9	(E) Excessive preoccupation with seeking drug reinforcers
Alcohol—positive consequences^b^	7	20	(E)
Smoking—nicotine dependence^c^	8	27	(E)
Smoking—emotional/sensory expectancies^c^	6	17	(E)
Separate from PROMIS^®^ Domains: PhenX Core Tier-1 Measures of Substance Abuse and Addiction Collection, as well as Core Tier-2 Measures whenever possible and appropriate^d^	Not applicable	Not applicable	Applicable to assessing substance use, demographics, and other clinical characteristics

*^a^For more information, see Ref. (9)*.

*^b^Applicable to all alcohol drinkers, daily alcohol drinkers, or non-daily alcohol drinkers*.

*^c^Applicable to all nicotine smokers, daily nicotine smokers, or non-daily nicotine smokers*.

*^d^For more information: *Notice Announcing Data Harmonization for Substance Abuse and Addiction via the PhenX Toolkit: Notice Number: NOT-DA-12-008 [Internet]*. Bethesda, MD: National Institutes of Health (US) (2016). Available from: http://grants.nih.gov/grants/guide/notice-files/NOT-DA-12-008.html, https://www.phenxtoolkit.org/index.php?pageLink=browse.core.tier1*.

*^e^Per the PROMIS website, regarding time to administer “each CAT takes 1–2 min. If you take three CATs, it will take 3–6 min to answer all the questions and get your report.” Examples of the PROMIS CATs may be found at https://www.assessmentcenter.net/ac1/Assessments/CATDemo*.

*^f^Mapping ASPIRE problem categories to the ANA domains*.

*ASPIRE problem categories A, S, and I map on to the ANA’s negative emotionality domain*.

*P and R map on to ANA’s executive function*.

*E maps on to ANA’s incentive salience*.

To conclude, Kwako et al. recently proposed an ANA, which includes a broad myriad of neuroscience-based functional domains associated with a SUD nosology together with a long battery. To maximize applicability among various clinical research situations and for collection as part of a patient registry or *via* electronic health record systems, it would be vital to tailor assessments and present only those pertaining to SUD risk categories or functional domains reported as being most salient to the research participant. Therefore, for deep-phenotyping purposes applicable to precision medicine research, a leaner, customizable assessment battery omitting less relevant measures, would be an important tool to advance research in this area.

## Author Contributions

UG undertook a review of the literature, conceived of this general commentary, and wrote and reviewed all drafts.

## Conflict of Interest Statement

The author declares that the research was conducted in the absence of any commercial or financial relationships that could be construed as a potential conflict of interest.
